# Monitoring disease activity of pollen allergies: What crowdsourced data are telling us^☆^

**DOI:** 10.1016/j.waojou.2022.100718

**Published:** 2022-11-14

**Authors:** Anna Schober, Linda Tizek, Emma K. Johansson, Agneta Ekebom, Jan-Erik Wallin, Jeroen Buters, Simon Schneider, Alexander Zink

**Affiliations:** a Technical University of Munich, School of Medicine, Department of Dermatology and Allergy, Munich, Germany; b Division of Dermatology and Venereology, Department of Medicine Solna, Karolinska Institutet, Stockholm, Sweden; c Department of Dermatology, Karolinska University Hospital, Stockholm, Sweden; d Department of Environmental Research and Monitoring, Swedish Museum of Natural History, Stockholm, Sweden; e Pollen Laboratory in Umeå Ltd, Umeå, Sweden; f Center Allergy & Environment (ZAUM), Member of the German Center for Lung Research (DZL), Technical University Munich/Helmholtz Center Munich, Munich, Germany

**Keywords:** Crowdsourcing, Epidemiological monitoring, Pollen, Public health surveillance, Rhinitis, Allergic

## Abstract

**Background:**

Pollen allergies are a major public health concern worldwide. An IgE-mediated systemic inflammatory response to pollen allergens causes symptoms of allergic rhinitis or even asthma. They have a significant impact on individual quality of life and cause high socioeconomic strain. The aim of this study was to examine the value of pollen allergy-related web search data for public health.

**Methods:**

An in-depth analysis of search volumes and contents, and their correlation with factors of disease activity such as rates of dispensed medicine and pollen concentration, was conducted. In this retrospective longitudinal study, Google Ads Keyword Planner was used to determine the internet search volume of terms related to pollen allergies across Germany and Sweden as a whole and in each of the 16 German federal states and 21 Swedish provinces between January 2017 and December 2020. This search volume was converted into searches per 100,000 inhabitants and categorized qualitatively.

**Results:**

A total search volume of 7405 searches per 100,000 inhabitants in Germany and 17,592 searches per 100,000 inhabitants in Sweden was observed, with the total yearly search volume increasing continually in both countries during the study period. Regional search volume correlated with antihistamine dispensation rates (ρ = 0.848–0.960) and pollen concentration (ρ = 0.566–0.922). While overall search interest was higher in Sweden, a higher interest in treatment options was identified in Germany.

**Conclusion:**

Internet websearch data is an excellent proxy for disease activity of allergic rhinitis. In the 4-year study period, the interest in pollen allergies has increased and there are unmet medical needs in both countries.

## Introduction

Seasonal allergies caused by pollen allergens are a major global public health concern,^1^ particularly as climate change increases the geographical spread and length of exposure to allergenic pollen.^2^ It has been estimated that 40% of Europeans are sensitized to pollen allergens.^3^

Pollen allergies lead to an IgE-mediated systemic inflammatory response to seasonal allergens like birch tree or grass pollen, typically causing allergic rhinitis (or “hay fever”), which is characterized by nasal congestion, rhinorrhea, itching of the nose, and sneezing.^4^ These symptoms have a significant impact on quality of life, as they often impair social life, sleep quality, and school or workplace productivity.^4^, ^5^, ^6^ Pollen allergies are also associated with other common diseases such as asthma, chronic hyperplastic eosinophilic sinusitis, nasal polyposis, and atopic dermatitis based on their shared type-2 immune responses.^4^

Antihistamines are the main symptomatic treatment for mild to moderate disease, with intranasal corticosteroids being used for more severe cases.^4^ Evidence suggests that causal treatment of symptomatic allergic rhinitis with allergen-specific immunotherapy can prevent exacerbations and progression to other diseases such as asthma.^7^ Effective diagnosis and treatment are crucial for adequately managing symptoms, improving quality of life, and reducing the socioeconomic costs of the disease. However, allergies are often underdiagnosed or trivialized and therefore not treated adequately, with many affected individuals lacking an official diagnosis from a health-care practitioner or appropriate medication.^8^, ^9^, ^10^ This indicates a gap between the provided healthcare and medical needs of a population.

To combat the public health issues inflicted by pollen allergies, new methods for monitoring airborne pollen and disease activity as well as for gaining an understanding of health-seeking behavior in a population are needed. A promising approach is the referral to crowdsourced data such as web search data as a tool for epidemiological surveillance.^11^^,^^12^ Previous studies using web search data on diseases have demonstrated the potential and value of search patterns for quantifying disease burden and have also given insight into health-seeking behaviors.^13^, ^14^, ^15^, ^16^

The aim of this study was to examine the value of pollen allergy-related web search data for public health applications by conducting an in-depth analysis of absolute regional search volumes and contents, seasonal trends, and correlations with environmental factors and disease activity as mirrored by antihistamine prescription rates.

## Methods

The Google Ads Keyword Planner was used to obtain web search data related to hay fever and pollen allergies across Germany and Sweden from January 2017 to December 2020. Germany and Sweden were chosen as together they cover a large north-south axis in Europe, making an analysis of search behavior across different vegetational and climatic zones possible. Additionally, a lot of public health information is publicly available in Sweden.

The Keyword Planner is originally aimed at optimizing advertisements, but it can equally be used for conducting scientific studies.^13^, ^14^, ^15^ With 70% of the population in Sweden and 57% of the population in Germany using the internet as a source of information for health purposes^17^ and more than 90% of German and Swedish inhabitants using Google as their main search engine,^18^ search data obtained from Google is expected to provide a comprehensive representation of the search behavior of these populations.

In this study, Google Ads Keyword Planner was used to identify the search terms (“keywords”) associated with the German and Swedish words for “hay fever” (German: Heuschnupfen; Swedish: hösnuva) and “pollen allergy” (German: Pollenallergie; Swedish: pollenallergi). Based on these 2 words, a list of relevant keywords and their monthly search volume from January 2017 to December 2020 was generated for each of the 16 German federal states and 21 Swedish counties as well as for each country as a whole. The language preference was set to each country's official national language and only searches made with a German or Swedish Internet Protocol address (IP) were included.

Since the study was based on publicly available search terms, institutional review board approval was not needed, and informed consent was not applicable.

All keywords were examined whether they explicitly referred to pollen allergies, hay fever, or allergies in general, and then classified accordingly into “specific” and “non-specific” keywords; non-specific keywords were excluded from the analysis. Specific keywords were further assigned to seven inductively created categories: (1) general (eg, “hay fever”), (2) allergy triggers (eg, “birch pollen allergy”), (3) symptoms (eg, “hay fever cough”), (4) stage of life (eg, “pollen allergy children”), (5) therapy (eg, “pollen allergy treatment”), (6) time of year (eg, “hay fever July”), and (7) other (eg, “hay fever cat”). Keywords that matched several criteria were assigned to multiple categories. Subsequently, the total search volume was calculated and descriptively analyzed.

To assess regional and seasonal differences in search behavior, the monthly search volume for each of the 16 German federal states and 21 Swedish counties was calculated and expressed as search volume per 100,000 inhabitants (Sources: Statistics Sweden, *Statistisches Bundesamt Deutschland*^19^). It was then investigated whether there was a correlation between the search volume and a monthly pollen index (sum of the pollen concentration of the most common allergenic pollen: alder, artemisia, birch, hazel, plantain, grasses, sorrel, oak, and ash), birch pollen concentration, climate data (monthly mean temperature, precipitation, and sunshine hours), and the monthly number of prescriptions of antihistamines. Swedish climate data was obtained from the Swedish Meteorological and Hydrological Institute (SMHI). The Swedish pollen data was provided for seven stations (Gävle, Visby, Eskilstuna, Stockholm, Umeå, Östersund, and Jönköping) by the Swedish Museum of Natural History, which coordinates the national pollen stations. The rates of antihistamines dispensed on prescription in Swedish pharmacies were accessed via Statistics Sweden. German weather data was obtained through the German weather service (“Deutscher Wetterdienst”, DWD^20^). Pollen concentration for Bavarian stations was provided by the Center of Allergy and Environment Munich (ZAUM); the monthly average of these pollen concentrations was used as a proxy for the airborne pollen in the federal state.

To describe the relationship between the investigated variables, SPSS version 25.0 (IBM Corp) was used to calculate the non-parametric Spearman correlation coefficient (ρ). A non-parametric test was used, as data for the variables were not normally distributed. Kruskal-Wallis one-way analysis of variance was used to determine regional differences in search volume per 100,000 inhabitants (standardized). *P*-values <0.05 were considered significant.

## Results

Overall, 1106 different search terms (“keywords”) were identified in Germany, of which 972 were specifically (87.9%) associated with pollen allergies. In Sweden, 336 keywords were identified, with 198 being specific keywords (58.9%). The specific keywords had a search volume of 6,158,860 (79.0% of total search volume) in Germany and 1,817,250 (80.9% of total search volume) in Sweden. After adjusting for the number of inhabitants, the relative search volume was 7405 searches per 100,000 inhabitants in Germany and 17,592 searches per 100,000 inhabitants in Sweden.

Considering the German federal states, the search volume per 100,000 inhabitants was found to be significantly higher (p < 0.001) in Hamburg (n = 15,436) and Bremen (n = 15,206) than in many other states like Baden-Wurttemberg (n = 8227), Bavaria (n = 7591), Brandenburg (n = 7163) and Saxony-Anhalt (n = 6977). In the Swedish counties, the search volume per 100,000 inhabitants was highest in Gotland (n = 28,985) and Västmanland (n = 23,546) and lowest in Norrbotten (n = 17,589) and Skåne (n = 17,072); however, the differences were not significant (p = 0.673).

**Fig. 1:**
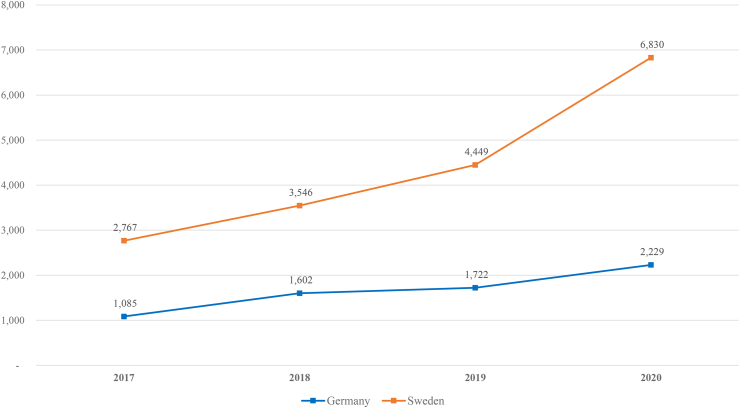
Total yearly search volume per 100,000 inhabitants from 2017 to 2020 in Germany and Sweden.

Fig. 1 shows the number of total annual searches, which increased every year in both countries. In 2017, a total of 1085 search queries were made per 100,000 inhabitants in Germany; this number was 2.05 times higher in 2020, when 2229 search queries were made per 100,000 inhabitants. In Sweden the number of search queries increased by a factor of 2.47 from a total of 2767 searches per 100,000 inhabitants in 2017 to 6830 searches per 100,000 inhabitants in 2020.

### Variation over the course of the year

**Fig. 2:**
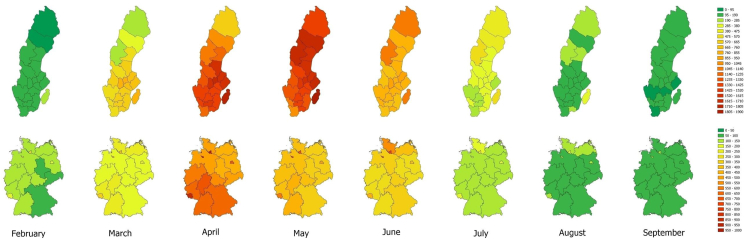
Average monthly search volume per 100 000 inhabitants in the German federal states and Swedish counties between 2017 and 2020.

The search volume showed noticeable variability over the year (See Fig. 2). In both countries, average monthly search volume was lowest in December (Germany: n = 24.5/100,000 inhabitants; Sweden: n = 35.8/100,000 inhabitants) and increased by 2225% (Germany) and 3617% (Sweden) until peaking in April and May. In Germany, monthly search volume was highest in April across all 4 years analyzed in the study, with 544.5 searches per 100,000 inhabitants on average. In Sweden, average search activity levels were similarly high in April (n = 1293.2/100,000 inhabitants) and May (n = 1296.3/100,000 inhabitants). Overall, April 2020 was the month with the highest search volume in both Germany (n = 760.4/100,000 inhabitants) and Sweden (n = 2068.1/100,000 inhabitants).

Fig. 2 shows the average monthly search volume in each county or federal state across the study period. In all German states except for Schleswig-Holstein, April was the month with the highest demand for information regarding allergies. In Schleswig-Holstein, average monthly search volume was highest in June. In Sweden, peaks were different in southern and northern counties. In southern counties such as Skåne and Halland, April was the month with the highest average search volume, whereas the average search volume peaked in May in northern counties such as Norrbotten, Västerbotten, Jämtland, and Västernorrland. Many other Swedish counties had similarly high levels of search volume in April and May.

### Correlation of search volume with environmental factors and antihistamine prescription rates

In the German states and Swedish provinces, various significant correlations were found between the regional search volume and pollen concentration, different climactic factors, and antihistamine prescription rates (Table 1).

**Table 1:** Range of the Spearman correlation coefficient (ρ) for search volume and different environmental factors in individual German states and Swedish provinces (lowest to highest)

	Germany	Sweden
Total pollen concentration	0.566^a^,^d^	0.820^a^ - 0.922^a^
Birch pollen concentration	0.456^a^,^d^	0.683^a^ - 0.818^a^
Mean temperature	0.408^a^ - 0.507^a^	0.357^b^ - 0.661^a^
Monthly precipitation	- 0.013^c^ - −0.406^a^	0.028^c^ - −0.408^a^
Antihistamine prescription rates	–	0.848^a^ - 0.960^a^

a P < 0.01.

b P < 0.05.

c Non-significant.

d Only Bavarian pollen data available.

In the Swedish counties, a strong correlation with the total pollen concentration was observed, which ranged from ρ = 0.820 (p < 0.01; Jämtland) to ρ = 0.922 (p < 0.01; Gotland). A high correlation between the relative search volume and birch pollen concentration was also detected, ranging from ρ = 0.683 to ρ = 0.818 (p < 0.01). In Bavaria, the search volume moderately correlated with the total pollen concentration (ρ = 0.566; p < 0.01) and birch pollen concentration (ρ = 0.456; p < 0.01).

Correlations between the monthly mean temperature and the search volume were detected, ranging from ρ = 0.357 (p < 0.01; Blekinge) to ρ = 0.661 (p < 0.01; Norrbotten) in Sweden and ρ = 0.408 (p < 0.01; Brandenburg) to ρ = 0.507 (p < 0.01; Bavaria) in Germany.

For precipitation, significant but weak or moderate negative correlations were observed in 8 Swedish counties; significant negative correlations in Germany were found in Saarland (ρ = −0.296; p < 0.05), Lower Saxony (ρ = −0.297; p < 0.05), and North-Rhine Westphalia (ρ = −0.406; p < 0.01).

The correlations between rates of antihistamines dispensed on prescription and search volume were very strong in all Swedish counties. The highest correlation was demonstrated in Stockholm (ρ = 0.960; p < 0.01); whereas, the lowest one was found in Kalmar (ρ = 0.848; p < 0.01).

### Search volume by categories

**Fig. 3:**
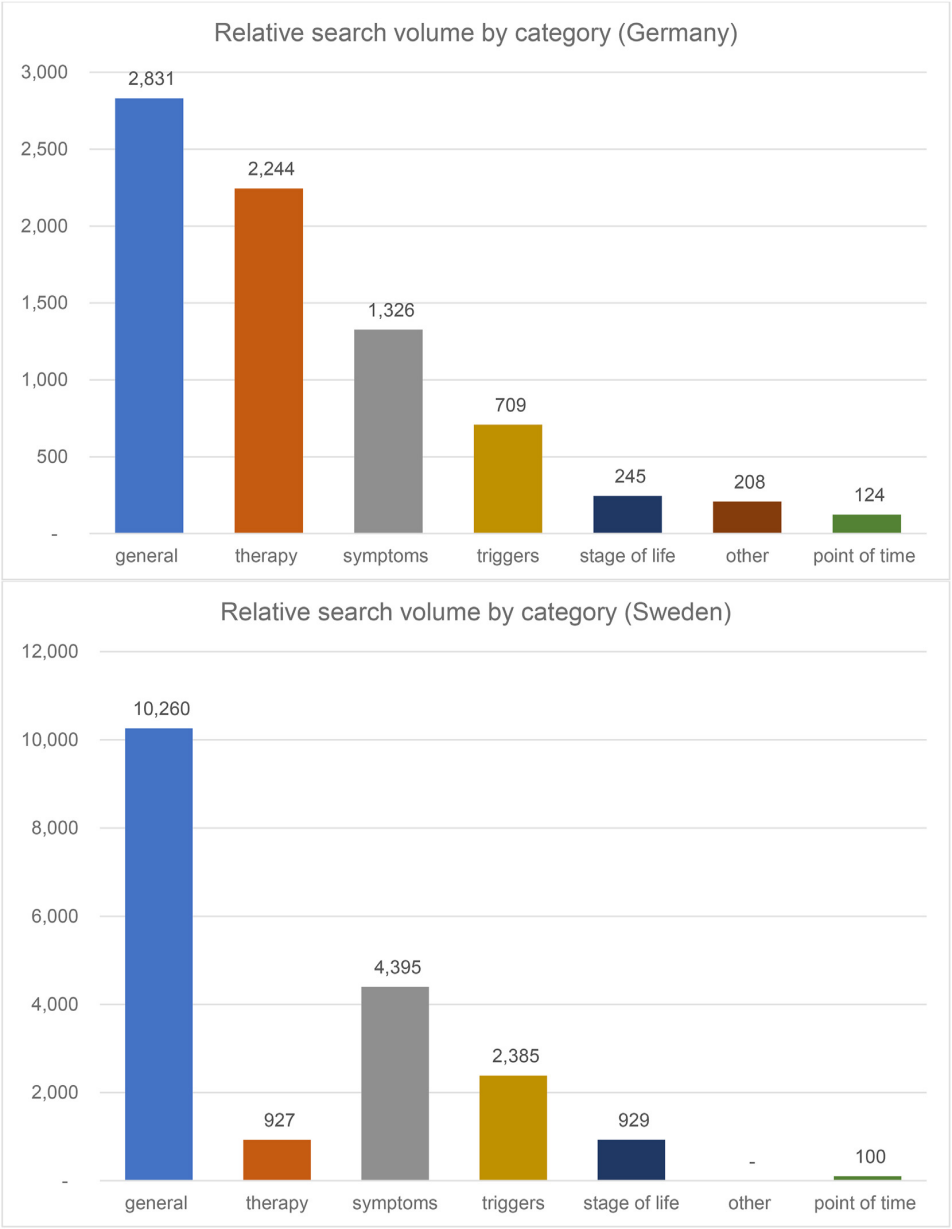
Relative search volume by category.

In Germany, most of the specific search queries were assigned to the general category (n = 2831/100,000 inhabitants ≙ 38.2%), therapy category (n = 2244/100,000 inhabitants ≙ 30.3%), and symptom category (n = 1326/100,000 inhabitants ≙ 17.9%) (Fig. 3). In 11 out of 16 federal states, such as Thuringia, Saxony-Anhalt, Saarland, and Bremen, the largest proportion of the specific search volume was therapy-related. About 25% of these therapy-related search queries specifically mentioned alternative medicine like homeopathy or home remedies.

In Sweden, most specific searches were general (n = 10,260/100,000 inhabitants ≙ 58.3%) followed by searches in the symptom category (n = 4395/100,000 inhabitants ≙ 25.9%) and triggers category (n = 2385/100,000 inhabitants ≙ 13.6%); the therapy-related search volume only accounted for 5.3% of the total specific search volume (n = 927/100,000 inhabitants), which was a notably smaller proportion of searches than that in Germany (Fig. 3). There were no comparable searches for home remedies or homeopathy.

## Discussion

In this study on pollen allergy-related search behavior in Germany and Sweden, a north-south axis regarding seasonality of search interest was observed and strong correlations of search volume with pollen concentration and antihistamine prescription rates were demonstrated. Additionally, differences in search volume and keyword topics were discovered in comparisons of the two countries, indicating potential unmet medical needs. The notably higher relative search volume in Sweden compared to in Germany may reflect a greater medical need regarding pollen allergies in Sweden.

Prior studies showed that the prevalence of allergic rhinitis was between 26.9 and 30.9% in Sweden,^21^^,^^22^ while in Germany a prevalence between 14.8 and 20.6% was reported.^23^^,^^24^ Although a direct comparison between the countries is difficult due to different definitions and methods in epidemiological studies, the substantially different levels of interest in this study support the observation that the disease has a higher prevalence in Sweden. The significantly higher relative search volume in the German city-states of Bremen and Hamburg may be attributed to the fact that allergies have a higher prevalence in urban areas than in rural areas.^25^

A further aspect to consider when comparing the 2 countries is the ambitious objective of the Swedish government regarding digitalization and e-health,^26^ which lead to telemedical services like digital appointments becoming increasingly common.^27^ Thus, the high number of search queries could also be linked to the higher relevance of the internet and digitalization in Swedish healthcare.

A continuous increase in search volume observed during a 4-year time period has also been reported in similar studies on web search data related to sarcoidosis, abdominal pain, and skin cancer,^28^, ^29^, ^30^ suggesting an overall increase in the importance of the internet for health information. However, the relative increase in search volume related to pollen allergy or hay fever observed in this study was higher than the relative increase in search volume for these other diseases, demonstrating an additional increased interest in the disease independent from general changes in search behavior. This may be explained by a growing burden of hay fever and pollen allergies, contrasting with epidemiological findings stating that their prevalence appears to have reached a plateau in recent years.^31^ Additionally, the COVID-19 outbreak in 2020 occurring simultaneously with the onset of the pollen season is likely to have caused another peak in interest in pollen allergies and their symptoms, as allergy symptoms can resemble those of COVID-19 infection.^32^^,^^33^

### Variation over the course of the year

Among the various pollen allergens in Europe, birch and grass pollen are the most relevant to consider in Germany and Sweden due to the high prevalence of sensitization against them as well as their potency.^3^^,^^34^ The flowering period of birch, which is the most dominant tree pollen allergen, typically starts at the beginning of April in central Europe and then progresses northwards, starting in May in Northern Europe.^3^ Grass pollen season starts in May and peaks in June.^3^

This flowering pattern was mirrored by search behavior in our study: April was the month with the highest search volume in Germany, coinciding with the peak of birch pollen season. Interestingly, in southern Swedish counties the average number of searches was also highest in April, while it was highest in May in northern counties, reflecting the northward progression and subsequent delay in pollen exposure. In both countries, search frequency remained high in June, which can be linked to the peak of the grass pollen season.

The relationship between pollen seasonality and frequency of related searches was confirmed by the strong correlation between regional search volume and corresponding pollen concentrations, which supports similar findings from other studies on internet search volume and allergies.^35^^,^^36^ The consideration of climactic factors like temperature and precipitation, and the correlations with search activity that were demonstrated in this study contribute to an understanding of the role that web search data can play in predicting and assessing the pollen season across a larger spatial and temporal dimension.

The high correlation between antihistamine prescription rates and internet search volume emphasizes the clinical relevance of these findings. Furthermore, these findings are consistent with those of a study examining the relationship between internet search behavior and real-world epidemiological data of pollen allergies in the United States. The study analyzed airborne pollen concentration and antihistamine sales data, which detected similarly high correlations as those in our study.^37^ The validity of our findings is further supported by a study from the United Kingdom that has found correlations between web search data on asthma and allergic rhinitis and classical epidemiologic surveillance data.^38^

Disease surveillance using crowdsourced information like web search data may present a cost-efficient approach to “patient-centered” care in medicine. As this data can alert to seasonal or regional medical needs in real time, it can contribute to an efficient and timely allocation of medical resources and improve adequate and purposeful public health interventions.

### Qualitative analysis

There was a considerable difference in the proportion of searches focusing on therapy in Germany and Sweden. This difference may suggest a higher demand for information on allergy therapy in Germany than in Sweden. On one hand, this may be indicative of a greater general need for information or skepticism regarding prescribed treatments. On the other hand, it could also indicate a high rate of self-medication, especially as a quarter of these searches were about alternative treatments. According to a German study by Muzalyova et al, 47% of patients treated themselves with over-the-counter medication without medical supervision.^10^ Additionally, studies have found that about 25–56% of individuals affected by allergic rhinitis do not have an official diagnosis from a healthcare practitioner.^8^^,^^9^^,^^39^

The fact that a quarter of therapy-related searches in Germany were about alternative medicine, whereas there was no comparable search volume in Sweden, reflects the different cultural attitudes towards alternative treatment options. The average use of consumable alternative medicines like homeopathy has been found to be two to six times higher in Germany than in Sweden.^40^^,^^41^ The belief that such medicine has fewer side effects and a “wish to try everything” have been reported as the most frequent reasons for using alternative medicine ^42^.

It is likely that the internet acts as a source of information on the possibilities for self-medication or usage of complementary medicine. These findings demonstrate the ability of internet search data to reflect different healthcare behaviors and cultural perspectives. An analysis of such data can be used to develop targeted public health campaigns and disease prediction models. These findings also underline the importance of trustworthy and high-quality online resources and digital services in healthcare, particularly as the internet becomes an increasingly important source of health information.^17^

## Limitations

There are some limitations to this study. Since Google Ads Keyword Planner does not provide information on user general demographics, subgroups could not be examined. Another limitation is that the monthly search volume is based on estimates from a private algorithm, and thus it is not possible to fully assess data precision. Increasing the temporal resolution might result in even more valuable data; this however is currently limited by the provider of the algorithm. Additionally, automatic completion of search terms may potentially lead to frequently searched terms being searched more often and less frequently searched keywords being neglected even further.

## Conclusion

In the four-year study period, the interest in pollen allergies has increased in the 2 countries, reflecting the increasing relevance of the internet for health information and a growing interest in pollen allergies. Based on our findings, internet search data appears to be an excellent proxy for disease activity of allergic rhinitis. As this cost-efficient data is available in real-time compared to data from conventional medical settings, it can provide valuable insight into current developments in disease activity. Combined with other influential factors like climate or geography, web search data can serve as a tool to identify and predict spatial and regional dimensions of the pollen season. Specific medical needs of a population can therefore be identified, and the timely allocation of medical resources and public health interventions can be optimized accordingly.

## Abbreviations

None used.

## Conflicts of interest

The authors declare that there are no conflicts of interest and no competing interests.

## Ethics approval

Since the study was based on publicly available search terms, institutional review board approval was not needed, and informed consent was not applicable.

## Funding

The study was funded by the Division of Dermatology and Allergy, Department of Medicine Solna, 10.13039/501100004047Karolinska Institutet, Stockholm, Sweden and the Department of Dermatology and Allergy, 10.13039/501100005713Technical University of Munich, Munich, Germany.

## Authors’ contribution

Conceptualization, project administration: AS, LT, AZ; data collection: LT, AZ, AS, AE, JB, JW; data preparation and analysis: AS, LT, SS; writing original draft: AS, LT, AZ, EJ; supervision, review, and editing: AS, LT, AZ, SS, JB, AE, JW, EJ.

## Authors’ consent for publication

All authors read and approved the manuscript and its publication.

## Availability of data and materials

Data and materials are available on request.

## Confirmation of unpublished work

This manuscript is original, has not been published before, is not currently being considered for publication elsewhere, and has not been posted to a preprint server.
